# Fractalkine regulation of microglial physiology and consequences on the brain and behavior

**DOI:** 10.3389/fncel.2014.00129

**Published:** 2014-05-13

**Authors:** Rosa Chiara Paolicelli, Kanchan Bisht, Marie-Ève Tremblay

**Affiliations:** ^1^Division of Psychiatry Research, University of ZurichZurich, Switzerland; ^2^Axe Neurosciences, Centre de Recherche du CHU de QuébecQuébec, Canada; ^3^Department of Molecular Medicine, Université LavalQuébec, Canada

**Keywords:** microglia, neurons, CX3CR1, fractalkine, development, synapses, neurogenesis, behavior

## Abstract

Neural circuits are constantly monitored and supported by the surrounding microglial cells, using finely tuned mechanisms which include both direct contact and release of soluble factors. These bidirectional interactions are not only triggered by pathological conditions as a S.O.S. response to noxious stimuli, but they rather represent an established repertoire of dynamic communication for ensuring continuous immune surveillance and homeostasis in the healthy brain. In addition, recent studies are revealing key tasks for microglial interactions with neurons during normal physiological conditions, especially in regulating the maturation of neural circuits and shaping their connectivity in an activity- and experience-dependent manner. Chemokines, a family of soluble and membrane-bound cytokines, play an essential role in mediating neuron-microglia crosstalk in the developing and mature brain. As part of this special issue on *Cytokines as players of neuronal plasticity and sensitivity to environment in healthy and pathological brain*, our review focuses on the fractalkine signaling pathway, involving the ligand CX_3_CL1 which is mainly expressed by neurons, and its receptor CX_3_CR1 that is exclusively found on microglia within the healthy brain. An extensive literature largely based on transgenic mouse models has revealed that fractalkine signaling plays a critical role in regulating a broad spectrum of microglial properties during normal physiological conditions, especially their migration and dynamic surveillance of the brain parenchyma, in addition to influencing the survival of developing neurons, the maturation, activity and plasticity of developing and mature synapses, the brain functional connectivity, adult hippocampal neurogenesis, as well as learning and memory, and the behavioral outcome.

## Introduction

Among the many strategies used by cells to communicate one with another, the repertoire of chemokines constitutes one of the most tightly regulated systems. Often, a particular cell type is uniquely expressing the ligand for a receptor that is selectively found on another cell type, thereby conferring a high degree of specificity to the ensuing signaling (Rossi and Zlotnik, 2000; Zlotnik and Yoshie, 2000; Allen et al., 2007). This is the case of fractalkine, a chemokine which signals directly from the producing neurons to their effector microglia, the only cells expressing its cognate receptor CX_3_CR1 in the healthy brain (Nishiyori et al., 1998; Schwaeble et al., 1998; Maciejewski-Lenoir et al., 1999; Hughes et al., 2002).

Fractalkine, also known as CX_3_CL1, is the only member of the ∂ (CX_3_C) chemokine family, characterized by the presence of 3 amino acidic residues (X_3_) localized between 2 cysteine residues, thus forming a disulphide bond, a CX3C motif, and also a transmembrane domain (Pan et al., 1997). The full-length fractalkine protein consists of 397 amino acids encoded by the *Cx3cl1* gene mapped on the chromosome 16 in human (Bazan et al., 1997; Pan et al., 1997) and of 395 amino acids encoded by the neurotactin gene mapped on the chromosome 11 in mouse (Rossi et al., 1998). Fractalkine is constitutively expressed at high levels by neurons, mostly in forebrain structures such as the hippocampus, amygdala, cerebral cortex, globus pallidus, striatum and thalamus, but also in the olfactory bulb, with almost no expression in the cerebellum, at the mRNA and protein levels in adult mouse *in situ* (Tarozzo et al., 2003). In the brainstem, a few scattered cells immunoreactive for fractalkine were initially depicted by Tarozzo and colleagues, while significant expression of the protein was subsequently observed *in situ* (Heinisch and Kirby, 2009; Ruchaya et al., 2012). Besides this neuronal expression, fractalkine mRNA and protein was also shown to be constitutively expressed by astrocytes, albeit at lower levels, in adult mouse, rat and human brain *in situ* (Hulshof et al., 2003; Sunnemark et al., 2005).

Fractalkine is a unique chemokine in that it exists in two different forms: a membrane-bound protein tethered to neuronal membranes by a mucine-like stalk (approximately 95 kDa), and a soluble factor released upon cleavage of its N-terminal chemokine domain (approximately 70 kDa) (Garton et al., 2001). Membrane-bound fractalkine has been proposed to act as an adhesion molecule, whereas the diffusible form works as an extracellular chemoattractant promoting cellular migration. This function is shared with other members of the chemokines family, commonly acting as “**chemo**tactic cyto**kines**” during innate and adaptive immunity. The name “chemokines” is precisely derived from this ability to mediate attraction of their responsive cells (Bazan et al., 1997; Comerford and McColl, 2011; Pan et al., 2011; Zlotnik and Yoshie, 2012).

Chemokine receptors belong to the family of G-protein coupled receptors (GPCR), showing the presence of 7 transmembrane helices connected by several intra- and extracellular loops, as well as N-terminal extracellular and C-terminal intracellular domains. The N-terminal extracellular domain is considered important for chemokine binding and receptor activation, while the C-terminal end is coupled to G-proteins, and is important for receptor signaling upon chemokine binding. Based on their primary amino acid sequence and the respective ligands that they bind, chemokine receptors are also classified into four subfamilies, i.e., CXCR, CCR, CR and CX3CR (Proudfoot et al., 2010). The fractalkine receptor CX_3_CR1 is a G_i_-protein coupled receptor encoded by the *Cx3cr1* gene, previously named V28, located on the chromosome 3 in human (Combadiere et al., 1995) and the chromosome 9 in mouse (Combadiere et al., 1998). The subunit protein Gi inhibits the production of cAMP, triggering a variety of intracellular second messengers including phosphoinositide 3-kinase (PI3K), protein kinase B (AKT) and nuclear factor kappa-light-chain-enhancer of activated B cells (NFκB), which are well-known for mediating a wide range of cellular functions, including apoptosis, proliferation, transcription and migration (Al-Aoukaty et al., 1998; Chandrasekar et al., 2003). CX_3_CR1 is ubiquitously expressed by monocytes, dendritic cells, and natural killer cells throughout the body (Imai et al., 1997; Combadiere et al., 1998; Harrison et al., 1998; Jung et al., 2000). Since these cells rarely infiltrate the brain parenchyma during normal physiological conditions, resident microglia are considered the only source of CX_3_CR1 expression and thus the only recipient of fractalkine signaling in the healthy brain (Jung et al., 2000; Mizutani et al., 2012).

In recent years, microglia were demonstrated to originate from yolk-sac derived progenitors infiltrating the brain during early embryonic development, with no subsequent contribution to their renewal from bone-marrow derived myeloid cells (Ginhoux et al., 2010; Mizutani et al., 2012; Kierdorf et al., 2013). Microglia were also shown to be extremely dynamic in their once presumed “resting” state, continuously surveying the brain parenchyma and contacting pre- and post-synaptic elements with their highly motile processes (Davalos et al., 2005; Nimmerjahn et al., 2005; Wake et al., 2009; Tremblay et al., 2010). Their physiological roles discovered so far comprise the elimination of supernumerary neurons and the maturation of synapses in the developing brain (Hoshiko et al., 2012; Cunningham et al., 2013; Lenz et al., 2013; Ueno et al., 2013), the regulation of neuronal and synaptic activity (Li et al., 2012; Pascual et al., 2012), the elimination of apoptotic newborn neurons generated in excess during adult hippocampal neurogenesis (Sierra et al., 2010), and the activity- and experience-dependent remodeling of neuronal circuits. Neuronal circuit plasticity is required for learning and memory processes in the developing and mature brain, where microglia contribute to both the formation and elimination of synapses (Tremblay et al., 2010, 2012; Paolicelli et al., 2011; Schafer et al., 2012; Parkhurst et al., 2013).

The CX_3_CR1-GFP knock-in mouse line where the fractalkine receptor gene has been replaced by a green fluorescent protein (GFP) reporter (Jung et al., 2000), represents one of the most important tools for studying microglial involvement in the healthy brain. All microglial cells abundantly express GFP, resulting in an exceptional fluorescent labeling of their complex arborization, from cell body to distal processes (Tremblay et al., 2010). Since the CX_3_CR1-GFP homozygous mice (CX_3_CR1^KO/KO^) are completely devoid of microglial CX_3_CR1, and therefore of fractalkine signaling, comparing the heterozygous (CX_3_CR1^KO/+^) used for imaging with the homozygous mice also provides a strategy for dissecting the molecular determinants of neuron-microglia communication in a non-invasive manner (Tremblay, 2011; Wolf et al., 2013). The CX_3_CR1-GFP heterozygous mice may be partially deficient in fractalkine signaling. Nevertheless, microglial morphology, dynamic surveillance (Nimmerjahn et al., 2005; Wake et al., 2009), dendritic spine turnover (Parkhurst et al., 2013), and microglial interactions with synaptic elements (Wake et al., 2009; Tremblay et al., 2010) were comparable *in vivo* between these CX_3_CR1-GFP heterozygous mice and the Iba1-GFP mice from Hirasawa and colleagues where CX_3_CR1 is not deleted (Hirasawa et al., 2005).

**Figure 1:**
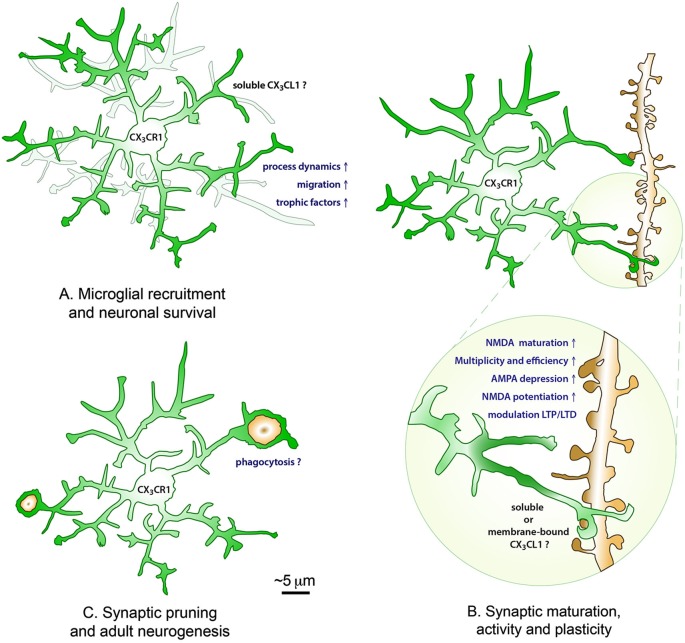
**The roles of CX_3_CL1-CX_3_CR1 interactions in the healthy brain**. During postnatal development, fractalkine signaling is promoting microglial recruitment to neuronal circuits by increasing their process dynamics and cellular migration, as well as modulating neuronal survival via the release of trophic factors **(A)**. During postnatal development and adulthood, soluble and/or membrane-bound fractalkine further contribute to the maturation, activity and plasticity of excitatory synapses by promoting the maturation of NMDA receptors (transition from GluN2B to GluN2A subunits), the multiplicity and efficiency of synaptic transmission, the depression of AMPA receptors and potentiation of NMDA receptors, and the modulation of LTP and LTD at the Schaffer collateral synapse in the hippocampus CA1 **(B)**, with lasting consequences on the brain functional connectivity, learning and memory, and the behavioral outcome. Additionally, fractalkine signaling might be influencing the developmental pruning of synapses and adult hippocampal neurogenesis by regulating microglial phagocytosis of synaptic elements and newborn apoptotic cells during normal physiological conditions **(C)**.

In the past few years, an extensive literature largely based on the CX_3_CR1-GFP line revealed that fractalkine signaling influences a broad spectrum of microglial physiological properties. Within this perspective, our focused review is dedicated to the emerging roles of fractalkine signaling in the regulation of microglial motility, as much as its consequences on neuronal survival, synaptic pruning, maturation, function and plasticity, hippocampal neurogenesis, the brain functional connectivity, learning and memory, and on the behavioral outcome (see Figure 1 for a schematic overview).

## Modulatory effects of fractalkine on microglial properties

Considering the crucial roles conferred by chemokines in the immune system, the CX_3_CL1-CX_3_CR1 axis was similarly expected to induce effector responses that are tightly adjusted to the homeostatic needs of the neuronal circuitry in the brain. Establishing microglial interactions with their dedicated neuronal subsets might be one example. The first evidence for such an important function comes from *in vitro* observations by Maciejewski-Lenoir and colleagues that fractalkine exerts a strong, dose-dependent migratory effect on cultured microglia derived from newborn rats (Maciejewski-Lenoir et al., 1999). Fractalkine application rapidly triggered changes in microglial activity, on a time scale of minutes, as revealed by the elevation of their intracellular calcium mobilization, which triggered a cascade of signaling leading to their cytoskeletal rearrangement and resulting in their migration. This response of microglia was diminished with antibodies against CX_3_CR1 or pharmacological inhibitors of the G_i_ subunit protein, supporting the functional involvement of CX_3_CL1-CX_3_CR1 interactions (Maciejewski-Lenoir et al., 1999).

Following these pioneering observations, Liang and colleagues further investigated the effects of CX_3_CR1 signaling on microglial dynamics *ex vivo*, by using time-lapse confocal imaging of retinal explants from adult mice. In these experiments, microglial processes were found to be significantly slower in the CX_3_CR1-GFP homozygous mice (CX_3_CR1^KO/KO^), either during basal condition or in response to focal laser-induced injury, compared with heterozygous controls (CX_3_CR1^KO/+^). Not only was microglial motility reduced in the CX_3_CR1 deficient microglia, but also their migration towards the site of injury, which was determined over minutes (Liang et al., 2009). Similarly, Zhang and colleagues have provided evidence that fractalkine signaling is implicated in the regulation of microglial migration *in vitro*, using microglia freshly isolated from the retina of newborn rats, co-cultured with a photoreceptor cell line. In this study, microglial migration quantified in a transwell chemotaxis assay was also found to be reduced following treatment with neutralizing anti-CX_3_CR1 antibodies. Conversely, the addition of recombinant full-length or soluble fractalkine resulted in an increased proportion of microglial cells moving towards the photoreceptors (Zhang et al., 2012).

In the developing hippocampus, Paolicelli and colleagues also reported a slower increase of microglial density in the CX_3_CR1^KO/KO^ mice versus CX_3_CR1^KO/+^ littermates, between postnatal days (P)8 and 28 in the CA1 region (Paolicelli et al., 2011). A similar situation was recently described by Audinat and colleagues in the somatosensory cortex, where microglial infiltration normally occurs over the first postnatal days. In this study, microglial numbers were found to be significantly diminished among the centers of the barrel cortex which represent the whiskers, when comparing the CX_3_CR1^KO/KO^ mice with wild-type littermates at P5, but this effect was transient as similar levels of microglial density were reached by P9 (Hoshiko et al., 2012). More recently, microglial colonization of the motor cortex duding early postnatal development was also shown to be impaired in the absence of fractalkine signaling, resulting in microglial accumulation within the subcortical white matter of the CX_3_CR1^KO/KO^ mice at P5 (Ueno et al., 2013). Considering that CX_3_CL1 expression is normally upregulated in the brain over the course of embryonic and postnatal maturation (Mody et al., 2001), these observations support a chemoattractant function of fractalkine signaling during normal development, particularly aimed at recruiting microglial cells to the relevant neuronal circuits, requiring specialized functional intervention during a period of intense activity-dependent remodeling.

## Consequences of fractalkine on neuronal circuit function and plasticity

### Survival of neurons

During normal development, microglia also contribute to the elimination of supernumerary neurons in various brain regions, including the cerebral cortex, hippocampus and cerebellum. The proposed mechanisms comprise the release of superoxide ions triggering apoptosis, the phagocytosis of non-apoptotic neural precursors and newborn neurons, and the release of trophic factors promoting neuronal survival (Marin-Teva et al., 2004; Wakselman et al., 2008; Cunningham et al., 2013; Ueno et al., 2013). Fractalkine signaling was recently involved in the latter mechanism. In particular, apoptotic neurons immunopositive for the cleaved-caspase 3 or detected with a TUNEL labeling of DNA fragmentation were more frequently encountered within the motor cortex of CX_3_CR1^KO/KO^ mice versus wild-type controls at P5 (Ueno et al., 2013). In this study by Ueno and colleagues, the insulin-like growth factor 1 (IGF-1) was further identified as a microglial factor preventing neuronal apoptosis. Interfering with IGF-1 signaling significantly increased the number of dying neurons in wild-type mice, using either intraventricular injection of a peptide blocking autophosphorylation of the IGF-1 receptor (H-1356), or a small hairpin (sh)RNA silencing gene expression of IGF-1. In the CX_3_CR1^KO/KO^ mice, mRNA expression of the IGF-1 binding protein 5 (IGFBP5) was also increased, and the free levels of IGF-1 were decreased, while injection of IGFBP5 promoted neuronal apoptosis (Ueno et al., 2013). These observations propose that CX_3_CL1-CX_3_CR1 interactions could serve to regulate the number of neurons during normal development, notably by promoting microglial release of trophic factors, even though the signaling cascade mediating microglial release of IGF-1 upstream of fractalkine signaling remains to be elucidated.

In a complementary manner, microglia were recently shown to promote neurogenesis and oligodendrogenesis in the developing rat brain, by releasing pro-inflammatory cytokines. Microglial release of interleukin (IL)-1β, IL-6, tumor necrosis factor (TNF)α and interferon (IFN)γ was found to regulate subventricular zone (SVZ) neurogenesis between P1 and P10. Systemic injection of the anti-inflammatory antibiotic minocycline, which is commonly used to reduce microglial activation, was accompanied by reduced levels of pro-inflammatory cytokines, and numbers of proliferating cells, neuronal and oligodendrocytic progenitors. In the somatosensory cortex, between P6 and P8, minocycline was recently shown to increase neuronal death and microglial activation (Arnoux et al., 2014), but in this study by Shigemoto-Mogami and colleagues, the effects of minocycline on reducing microglial activation were confirmed by a strong reduction in activation markers such as CD11b and CD68. Interestingly, IGF-1 levels were not affected, thus suggesting that this signaling pathway is not regulating the effects of microglia on neurogenesis in the SVZ during the first postnatal days (Shigemoto-Mogami et al., 2014).

### Microglial pruning of synapses

In the healthy brain, microglial cells also participate to the remodeling of neuronal circuits, an activity- and experience-dependent process required for learning and memory, and proper behavioral outcome, that is particularly exacerbated during postnatal development, but still persists throughout adulthood and normal aging. So far, microglial contacts with pre-synaptic axon terminals and post-synaptic dendritic spines, sometimes accompanied by their phagocytic engulfment and elimination, have been documented in the cerebral cortex, hippocampus and thalamus using a combination of cutting-edge microscopy techniques: transcranial two-photon *in vivo* imaging (cerebral cortex), electron microscopy with immunostaining and 3D reconstructions (cerebral cortex, hippocampus, thalamus), and stimulated-emission depletion confocal microscopy (hippocampus) (reviewed in Tremblay, 2011; Kettenmann et al., 2013). Using these techniques, phagocytic inclusions identified as synaptic elements, based on their ultrastructural features (i.e., 40 nm synaptic vesicles and post-synaptic densities) or immunoreactivity for the synaptic markers vesicular glutamate transporter 2 (Vglut2), post-synaptic density protein 95 (PSD95), and synaptosomal-associated protein 25 (SNAP25), were visualized inside of microglial cell bodies and processes (Tremblay et al., 2010; Paolicelli et al., 2011; Schafer et al., 2012).

The involvement of fractalkine signaling in mediating the elimination of synapses during normal development was particularly addressed by comparing the CX_3_CR1^KO/KO^ mice with CX_3_CR1^KO/+^ littermates in the hippocampus CA1 during the first postnatal weeks. In the study by Paolicelli and colleagues, the decrease in microglial number was also found to be accompanied by a transient increase in dendritic spine density on the pyramidal neurons apical dendrites of the same region, thus indicating a possible role for fractalkine signaling in mediating microglial pruning of dendritic spines (Paolicelli et al., 2011). However, it remains to be elucidated whether fractalkine signaling exclusively instructs the recruitment of microglial cells to the proximity of maturing dendrites, or, in addition, also contributes to directly regulating microglial pruning itself.

In parallel, the work of Schafer and colleagues has shown that microglial pruning of synaptic elements is determined during postnatal development by the microglial complement 3 receptor (C3R), since microglial phagocytosis of axon terminals was significantly reduced *in situ* in the visual thalamus of C3R knockout mice at P5 and P20, resulting in a sustained impairment of synaptic density until P32–P35 at least (Stevens et al., 2007; Schafer et al., 2012). The subsequent work of Linnartz and colleagues additionally revealed that the classical complement cascade becomes recruited by the changes in neuronal glycocalyx *in vitro*. In particular, desialylated (i.e., lacking the terminal sugar residue sialic acid) neurites were found to be preferentially phagocytosed by microglial cells in primary culture via a CR3-dependent mechanism (Linnartz et al., 2012), but the molecular relationships between fractalkine signaling and the classical complement pathway remain unknown at this early stage of investigation in the field.

### Functional maturation of synapses

A role for fractalkine signaling in modulating the postnatal development of neuronal circuits was also lately proposed by the work of Hoshiko and colleagues. In this study, the delayed infiltration of microglial cells in the barrel cortex was accompanied by defects of synaptic maturation. Electrophysiological analyses in acute thalamocortical slices from CX_3_CR1^KO/KO^ mice versus wild-type littermates revealed that CX_3_CR1 deletion affects the functional maturation of postsynaptic glutamate receptors which normally occurs at thalamocortical synapses in the first 2 postnatal weeks. During the first postnatal week, NMDA receptors contain predominantly GluN2B subunits, replaced by GluN2A subunits by the end of the second postnatal week to increase the efficiency of synaptic transmission. Considered as an index of synaptic maturation, this switch is associated with faster kinetics of the NMDARs-mediated excitatory postsynaptic currents (EPSC). This transition from GluN2B to GluN2A subunits was precisely delayed in the CX_3_CR1 knockout mice. In particular, the decay time of the NMDAR-mediated responses was found to be significantly reduced at P9-10 in the CX_3_CR1^KO/KO^ mice compared with wild-type littermates, using single-cell recordings. However, the defect was transient, without any remaining difference in the decay time of NMDAR-mediated responses at P27–P33 (Hoshiko et al., 2012).

In addition, Zhan and colleagues recently reported differences in the regulation of EPSCs in the CX_3_CR1^KO/KO^ mice, by using single-cell recording at the Schaffer collateral synapse in acute hippocampal slices (Zhan et al., 2014). Both the miniature EPSCs (mEPSC) and spontaneous EPSCs (sEPSCs) were investigated. Since mEPSCs are recorded in presence of tetrodotoxin (TTX), a sodium channel blocker that prevents action potential-induced currents, they constitute a measure of post-synaptic currents induced by random release of synaptic vesicles. On the other hand, sEPSCs reflect currents triggered by an action potential, and confer a measure of synaptic efficiency. The amplitude of sEPSC arising from the action potential-dependent release of synaptic vesicles normally increases over the course of postnatal development, as functional synapses become increasingly formed and neuronal networks properly interconnected, a process named synaptic multiplicity. On the contrary, the amplitude of mEPSCs remains relatively constant across the same age interval. The ratio between sEPSCs and mEPSCs increases as neural circuits develop, and can be considered as an index of neural circuit maturation (Hsia et al., 1998). In littermate wild-type mice, the amplitude of sEPSCs was found to be significantly larger than mEPSCs both at P15 and P40. In CX_3_CR1 knockout mice, however, no significant difference was observed between action potential- (sEPSCs) and non-action potential-dependent (mEPSCs) currents at P15 and later at P40, suggesting reduced synaptic multiplicity and less efficient post-synaptic transmission when microglial function is compromised. With the previous observations, these recent findings support a crucial role for fractalkine signaling in regulating the maturation of excitatory synapses during normal development.

### Synaptic transmission

In line with these observations, several studies have recently revealed modulatory effects of fractalkine signaling on the electrophysiological properties of excitatory synapses in acute hippocampal slices. Ragozzino and colleagues have first reported that fractalkine negatively modulates the AMPA receptors-dependent component of glutamatergic transmission at the Schaffer collaterals synapse. In this study, the application of fractalkine induced a sustained, dose-dependent reduction of the evoked EPSC amplitude in the CA1 region of acute hippocampal slices from P14–P22 mice and rats. This depression of EPSC was not observed in slices from CX_3_CR1^KO/KO^ mice, and specifically depended on AMPA receptors function, being elicited by AMPA during the silencing of endogenous synaptic transmission with TTX and bicuculline (an antagonist of GABA_A_ receptors), and suppressed by the AMPA and kainate receptors blocker 6,7-dinitroquinoxaline-2,3-dione (DNQX). In contrast, the NMDA receptors blocker D-amino-5-phosphonovaleric acid (D-APV) produced no effects (Ragozzino et al., 2006). Bertollini and colleagues similarly observed that fractalkine induces a sustained but reversible depression of field excitatory postsynaptic potentials (fEPSP), reflecting the postsynaptic response to the stimulation of Schaffer collaterals recorded in a population of neurons, in the CA1 region of acute slices derived from 21–29-day old mice. These effects were absent in slices from CX_3_CR1^KO/KO^ mice, and further suppressed in wild-type mice after application of a CX_3_CR1 blocking antibody (Bertollini et al., 2006). Nevertheless, the mechanisms by which neuron to microglia crosstalk might contribute to these effects of fractalkine signaling on glutamatergic synapses function still remain unknown.

In this context, recent studies have suggested a possible involvement of microglial release of adenosine, not only in mediating the depression of AMPA receptors function, but also the potentiation of NMDA receptors function. A role for the purine nucleoside adenosine was first proposed by the work of Piccinin and colleagues, showing that fractalkine causes a reversible depression of EPSCs in acute hippocampal slices from 14–20 days old mice, an effect that is prevented by treatment with adenosine deaminase, an enzyme that irreversibly converts adenosine to the related nucleoside iosine, and abolished by the specific AR3 antagonist MRS1523, contrarily to the AR1 (DPCPX) and AR2 (SCH58261) antagonists. These observations were corroborated by additional findings that fractalkine induces EPSC depression in acute slices from AR1 and AR2 knockout mice, but not in slices from AR3 knockout mice (Piccinin et al., 2010). More recently, Scianni and colleagues further proposed that fractalkine-induced release of adenosine increases the amplitude of fEPSPs resulting from the stimulation of Schaffer collaterals, by acting on its NMDA dependent component specifically. In this study, the effects of fractalkine on the fEPSP were found to be abolished by the selective antagonist of NMDA receptors 5,7-dichlorokynurenic acid (DCKA), the specific AR2 blocker SCH58261, and in acute slices from AR2 knockout mice, thus suggesting a role for AR2 in mediating these effects. Upon fractalkine stimulation, a significant increase in the concentration of D-serine, a co-agonist of NDMA receptors, was also measured by mass spectrometry analysis in the extracellular medium of microglia and astrocyte primary cultures. Microglial (or astrocytic) release of D-serine might therefore potentiate NMDA receptors function downstream of fractalkine signaling (Scianni et al., 2013).

### Synaptic plasticity

How fractalkine-induced alterations of postsynaptic glutamatergic responses modulate the propensity for long-term synaptic plasticity was also recently investigated. In particular, Maggi and colleagues have used fEPSP recordings at the Schaffer collaterals synapse to reveal that fractalkine significantly inhibits long-term potentiation (LTP) when administered during the critical period for induction, in acute slices from 6–8 weeks old wild-type mice. This impairment of LTP was however absent in slices from CX_3_CR1^KO/KO^ mice, and additionally found to require the activation of AR3 receptors as it was prevented by the selective antagonist MRS1523 (Maggi et al., 2009), in agreement with the previous findings. Nevertheless, subsequent studies using CX_3_CR1^KO/KO^ mice to explore the effects of fractalkine signaling on long-term plasticity, in acute slices from the hippocampus CA1 region, reported a complete absence of LTP (Rogers et al., 2011), or a more sustained LTP than in wild-type littermates, when LTP was induced by a weak stimulation protocol (Maggi et al., 2011). This apparent discrepancy warranting further investigation might result from differences in the ages, diet and housing conditions of the animals, or electrophysiological preparation and stimulation protocols between studies. Lastly, Paolicelli and colleagues also examined the influence of fractalkine signaling on the induction of long-term depression (LTD) in the CX_3_CR1^KO/KO^ mice compared to wild-type littermates, reporting no difference between genotypes during adulthood (P40), despite a significant enhancement of LTD in the CX_3_CR1^KO/KO^ at P13 (Paolicelli et al., 2011), thus suggesting the possibility that only LTP could be modulated by fractalkine signaling in the mature healthy brain.

### Adult hippocampal neurogenesis

Neurogenesis continues throughout life in the hippocampus, where it was demonstrated to be necessary for synaptic plasticity, including the induction of LTP and LTD, as well as classical eye blink and fear conditioning, memory retention in spatial learning tasks, and encoding of overlapping input patterns (Sierra et al., 2014). Contributing to these processes, microglia have been shown to regulate the neurogenic cascade during normal physiological conditions, by their phagocytic elimination of apoptotic newborn cells, an efficient process that is undeterred by increased age or inflammatory challenge (Sierra et al., 2010). Conversely, microglial physiological properties such as their proliferation, migration and phagocytosis were recently found to be modulated by soluble factors, such as vascular endothelial growth factor (VEGF) released by neuronal progenitor cells (NPCs) when implanted in the striatum of adult mice *in vivo*, as supported by the work of Mosher and colleagues proposing that microglia are not only influencing neurogenesis, but may also be regulated by newborn cells (Mosher et al., 2012).

With respect to the involvement of fractalkine signaling, Bachstetter and colleagues first revealed that the density of proliferating, newly generated cells is significantly reduced in the subgranular zone (SGZ) of 4 month old CX_3_CR1^KO/KO^ mice compared with CX_3_CR1^KO/+^ littermates. Conversely, chronic treatment with fractalkine was found to promote neurogenesis in aged (22 months old) but not young (3 months old) or middle-aged rats (12 months old), while an antagonist of CX_3_CR1 produced opposite effects in young, middle-aged and old rats (Bachstetter et al., 2011). In a follow-up study, comparing CX_3_CR1^KO/KO^ and CX_3_CR1^KO/+^ mice with wild-type littermates further revealed that adult hippocampal neurogenesis is regulated by fractalkine signaling in a gene-dose dependent manner, with intermediate levels of neurogenesis measured in the heterozygous mice (Rogers et al., 2011). Supporting these results, Maggi and colleagues also described a reduced adult hippocampal neurogenesis in the SGZ of 13 to 14 weeks old CX_3_CR1^KO/KO^ mice, versus wild-type littermates, and further revealed that neurogenesis is positively regulated by chronic environmental enrichment, even in the absence of CX_3_CR1 (Maggi et al., 2011). Nevertheless, the cellular and molecular mechanisms which are mediating these effects downstream of fractalkine signaling remain unknown.

### Functional connectivity

Lastly, Zhan and colleagues have recently shown that CX_3_CR1^KO/KO^ mice display a lasting impairment of synaptic connectivity into adolescence, in the hippocampus CA1 region. In particular, quantifying at the ultrastructural level the density of multi-synaptic boutons, i.e., axon terminals making synapses with 2 dendritic spines, revealed a significant reduction in the knockout mice compared to wild-type littermates at P40. These data support the hypothesis that in the absence of fractalkine signaling, compromised neuron-microglia interactions are affecting the efficiency of synaptic transmission (Zhan et al., 2014) despite a normalized density of dendritic spines which was observed by the same group in adult animals (Paolicelli et al., 2011). In addition, local field potentials (LFPs) were investigated by Zhan and colleagues by performing *in vivo* recordings at P40. LFPs inform about the overall levels of electrical activity measured in a certain volume, resulting from the sum of synaptic activity. As a measure of long-range connectivity, coherence spectra of the LFPs measured locally were calculated, under the premise that high coherence values reflect strongly connected structures. By implanting electrodes within different brain regions simultaneously, a significant decrease in the coherence between the hippocampus and the prefrontal cortex was found in CX_3_CR1^KO/KO^ adult mice, compared to wild-type littermates, thus reflecting a decrease in functional connectivity. Interestingly, the coherence between the hippocampus and the prefrontal cortex was also investigated in behaving mice, during bouts of social investigation, revealing a significant increase in coherence following the onset of social investigation in wild-type mice, but not in CX_3_CR1 knockouts. In line with these results, the global functional connectivity assessed by functional magnetic resonance imaging (fMRI) showed a significant reduction in the connectivity across brain regions, becoming particularly evident for distant regions, in the CX_3_CR1^KO/KO^ mice versus wild-type controls (Zhan et al., 2014). These recent findings are complementing one another in supporting a fundamental role for CX_3_CL1-CX_3_CR1 interactions in mediating the development and plasticity of neuronal circuits, at the molecular, cellular and neuronal circuit levels.

## Consequences on learning, memory, and behavior

At the behavioral level, Rogers and colleagues also reported deficits in different forms of learning and memory in 3 month old CX_3_CR1^KO/KO^ mice, in parallel with the alteration of LTP. In particular, motor learning was found to be compromised in the CX_3_CR1 knockouts, compared to wild-type littermates, using standard rotarod training (Rogers et al., 2011). Locomotor and exploratory activity was however similar between the two genotypes, when assessed in the open field test, and no difference was observed in anxiety behavior, measured by the elevated plus maze. In addition, associative learning and memory was altered in a standard fear-conditioning paradigm: after a similar freezing behavior in the training session, knockout mice failed to display associative learning (i.e., reduced freezing) when tested 24 h later in the same environment (Rogers et al., 2011). On the contrary, mice exposed to conditioning in a novel environment displayed a similar freezing behavior across genotypes, probably underlying hippocampal-specific deficits in cognition, considering that context-specific types of associative memory predominantly depend on the hippocampus (Sousa et al., 2006; Crawley, 2008). The CX_3_CR1 knockouts also displayed deficits when performing the water maze test, thus further supporting a role for fractalkine signaling in modulating hippocampal-dependent learning and memory. These effects could be mediated by microglial release of the pro-inflammatory cytokine IL-1β since intrahippocampal infusion of its antagonist IL-1ra significantly reversed the deficits in cognitive function measured in the CX_3_CR1 knockouts (Rogers et al., 2011).

However, Maggi and colleagues revealed that the CX_3_CR1^KO/KO^ mice are not significantly different from aged-matched wild-type controls in their ability to learn the water maze task, when they are tested at 13 to 14 weeks of age, even though they fail to perform better following prolonged exposure to an enriched environment, compared with standard housing conditions. Since environmental enrichment also failed to increase LTP, as well as adult hippocampal neurogenesis in parallel experiments, it has been proposed that CX_3_CR1 deficiency could increase hippocampal plasticity as well as spatial memory, thereby blunting the potentiating effects of the environmental enrichment (Maggi et al., 2011). Here again, the discrepancy between studies is unclear, possibly resulting from differences in the animals or behavioral paradigms and analyses.

Interestingly, social interaction was also found to be altered in the CX_3_CR1^KO/KO^ mice, both early in life and into adulthood. Impaired social behavior was observed in juvenile mice, displaying no preference for their own mother over an inert stimulus, as assessed by the homing test. However, no impairment in performing the novel object recognition test was observed in the same mice. Similarly, adult CX_3_CR1 knockout mice tested in a standard three-chamber apparatus failed to display significant interest towards a sex-matched social stimulus, compared to wild-type controls (Zhan et al., 2014). No deficit in responding to social olfactory cues was reported in these mice, suggesting that the impairment observed in social behavior was due to reduced social motivation, rather than difficulties with the discrimination of social cues. Additionally, increased grooming behavior was reported in adult CX_3_CR1 knockout mice, when tested in a novel cage for 10 min, suggesting a propensity for increased repetitive behavior, particularly triggered under stressful conditions (Zhan et al., 2014).

## Conclusion

In recent years, a combination of imaging, electrophysiology, and behavioral analyses performed in the CX_3_CR1^KO/KO^ mice, versus CX_3_CR1^KO/+^ or wild-type littermates, have revealed that CX_3_CL1-CX_3_CR1 signaling crucially regulates the development and plasticity of neuronal circuits, with functional consequences on the brain connectivity, adult hippocampal neurogenesis, learning and memory, and the behavioral performance. Since microglia are the only cell type expressing CX_3_CR1 in the healthy brain, they might be crucially involved in every one of these processes shown to be influenced by fractalkine signaling during normal physiological conditions: the survival of newborn neurons, the maturation and elimination of synapses, the regulation of synaptic transmission, long-term synaptic plasticity, and adult hippocampal neurogenesis. Nevertheless, the microglial effector functions which are precisely recruited, the molecular mechanisms acting downstream of CX_3_CL1-CX_3_CR1 interactions, much as the respective contributions of soluble versus membrane-bound fractalkine to these essential processes of normal physiology remain to be elucidated.

## Conflict of interest statement

The authors declare that the research was conducted in the absence of any commercial or financial relationships that could be construed as a potential conflict of interest.
